# Efficacy of a First Course of Ibuprofen for Patent Ductus Arteriosus Closure in Extremely Preterm Newborns According to Their Gestational Age-Specific Z-Score for Birth Weight

**DOI:** 10.1371/journal.pone.0124804

**Published:** 2015-04-13

**Authors:** Doriane Madeleneau, Marie-Stephanie Aubelle, Charlotte Pierron, Emmanuel Lopez, Juliana Patkai, Jean-Christophe Roze, Pierre-Henri Jarreau, Geraldine Gascoin

**Affiliations:** 1 Department of Neonatal Medicine of Port-Royal, Groupe Hospitalier Cochin-Broca-Hôtel Dieu, APHP, Paris, France; 2 Department of Neonatal Medicine, Nantes University Hospital, Nantes, France; 3 Department of Neonatal Medicine, Angers University Hospital, Angers, France; The Ohio State Unversity, UNITED STATES

## Abstract

**Objective:**

Therapeutic strategies for patent ductus arteriosus (PDA) in very preterm infants remain controversial. To identify infants likely to benefit from treatment, we analysed the efficacy of a first course of ibuprofen in small-for-gestational age (SGA) newborns.

**Study design:**

This single-centre retrospective study included 185 infants born at 24+0–27+6 weeks of gestation with haemodynamically significant PDA, who were treated by intravenous ibuprofen (Pedea): 10 mg/kg on day one and 5 mg/kg on days two and three. Birth weight and gestational age (GA) were analysed with reference to the standard deviations from the Olsen growth curve to define GA-specific Z-scores for birth weights. The efficacy of treatment was evaluated by echocardiography 48 hours after the last dose of ibuprofen. The primary outcome was failure of the first course of ibuprofen associated in a composite criterion with the most severe outcomes.

**Results:**

The risk of treatment failure increased according to a continuous gradient in SGA neonates. A higher risk was observed on multiple regression analysis (crude OR: 3.8; 95% CI [1.2–12.3] p = 0.02; adjusted OR: 12.8; 95% CI [2.3–70.5] p=0.003).

**Conclusion:**

There is a linear relationship between infant birth weight and PDA treatment: the failure rate of a first course of ibuprofen increases with increasing degree of growth restriction.

## Introduction

The ductus arteriosus closes spontaneously in most healthy term newborns during the first three days after birth. However, more than two-thirds of infants delivered before 28 weeks of gestation or with a birth weight <1750 g develop patent ductus arteriosus (PDA), a heart condition that requires either pharmacological or surgical closure of the ductus arteriosus [1]. In Europe, most neonatal intensive care units use ibuprofen, a non-selective cyclooxygenase inhibitor, for patent ductus arteriosus closure. Small-for-gestational age (SGA) infants are at increased risk of short-term neurological and respiratory complications [2,3] and present an increased rate of PDA [4]. In 35% of cases, infants are born SGA as a result of uteroplacental insufficiency and chronic hypoxia. Intrauterine growth restriction *per se* increases mortality and morbidity by increasing the newborn’s prematurity [5], and the haemodynamic consequences of PDA are observed more frequently and earlier in these patients [6]. However, the group of preterm infants likely to benefit from PDA treatment, the timing of treatment, and the best therapeutic strategy are still a matter of debate [7,8]. To identify this population, various studies have examined the effect of birth weight [9] and gestational age (GA) [10]; however, to the best of our knowledge, no studies have investigated the effect of treatment according to birth weight standard deviation.

We conducted a single-centre retrospective study to analyse the success of a first course of ibuprofen to close significant PDA in infants born 24+0–27+6 weeks of gestation according to their degree of intrauterine growth restriction.

## Patients and Methods

### Study population

All live newborns admitted to the Cochin-Port Royal Hospital neonatal intensive care unit (Paris, France) between 1^st^ January 2005 and 31^st^ December 2009 were eligible for this study. The Institutional Review Board (Comité de Protection des Personnes Ile de France III) stated: “This research was found to conform to generally accepted scientific principles and research ethical standards and to comply with the laws and regulations of France, where the research was performed.” Written informed consent from the patients or parents was unnecessary for this retrospective study. Inclusion criteria were: GA of 24+0–27+6 weeks, echocardiographic evidence of haemodynamically significant left-to-right shunting across the PDA, and initiation of at least one course of intravenous ibuprofen (Pedea®). Echocardiographic criteria indicating the need for PDA treatment were ductus arteriosus diameter >1.5 mm and at least two of the following criteria: left atrial-to-aortic root ratio >1.5, pulsatile flow in the ductus arteriosus, or retrograde/absent diastolic flow in the anterior cerebral artery or descending aorta. Exclusion criteria were: major congenital malformations including congenital heart defects other than PDA, genetic or chromosomal abnormalities, death before determining ductus arteriosus status, and PDA in a clinical setting that precluded ibuprofen therapy (severe haemodynamic failure requiring vasoactive drugs; necrotising enterocolitis; intestinal bleeding; grade 3 or 4 intraventricular haemorrhage; right-to-left shunt on echocardiography; other signs of pulmonary hypertension, such as serum creatinine >120 μmol/L or platelet count <50,000/mm^3^; and clotting disorders).

### Data collection

All medical data were collected retrospectively from medical records. Medical records were compiled prospectively as part of routine neonatal care and were regularly validated by the clinical team. Birth weights were expressed in relation to GA in terms of standard deviations (SD) from Olsen growth curves [11]. Z-scores were divided into three groups: birth weight Z-score > -0.5 SD, birth weight Z-score -0.5 – -1.5 SD, and birth weight Z-score < -1.5 SD. Data concerning neonatal outcomes before discharge such as intrauterine infection, duration of endotracheal intubation and high frequency oscillatory ventilation, necrotising enterocolitis (grade 3 and 4 of Bell’s classification), grade 3 and 4 intraventricular haemorrhage (Papile classification), periventricular leukomalacia, bronchopulmonary dysplasia and death were also collected. The following treatment data were collected: age at initiation of treatment, number of courses of ibuprofen initiated (some courses were discontinued), discontinuation of the first course of ibuprofen, use of dopamine or furosemide during ibuprofen therapy, and patent ductus arteriosus surgery.

### Study design

The management of PDA remained the same throughout the study period in our unit. Infants born before 28 weeks of gestation were routinely assessed by echocardiography during the first 48 hours of life (earlier if they presented clinical signs of PDA). Intravenous ibuprofen was administered in three doses at 24-hour intervals: 10 mg/kg on day one and 5 mg/kg on days two and three. Echocardiography was performed before the first dose of ibuprofen and 48 hours after the last dose according to the unit’s standard policy.

If the first course of ibuprofen failed and the patient presented no contraindications to ibuprofen, a second course of ibuprofen was administered at the same doses. In the presence of a contraindication to ibuprofen, or if after failure of the second course of ibuprofen, PDA was treated surgically.

### Short-term outcomes

The primary outcome was failure of the first course of ibuprofen associated with the most severe outcomes in a composite criterion: surgery, necrotising enterocolitis, intestinal perforation, and dopamine and furosemide treatment during the first course of ibuprofen. Success of the PDA closure was defined as no flow in the ductus arteriosus on echocardiography performed 48 hours after the last dose of ibuprofen.

### Statistical analysis

Obstetric and neonatal characteristics were initially studied as a function of birth weight across the GA spectrum. The relationship between the characteristics of ibuprofen therapy and the degree of growth restriction was also studied. For descriptive univariate analyses, all statistical tests were two-tailed, and p<0.05 was considered significant. Statistical analysis was designed to identify potential confounders in the relationship between ibuprofen efficacy and growth restriction. This relationship was studied by using a logistic regression model, adjusted for obstetric and neonatal variables selected in the univariate analyses. The first step of our model considered only treatment failure and degree of growth restriction defined by GA-specific Z-scores. This basic relationship was expressed in terms of a crude OR and did not include any potential confounders. The next step analysed the same relationship and gradually included the confounders derived from the univariate analyses described above in order to determine whether the relationship between failure of the first course of ibuprofen and growth restriction was independent of potential confounders. Associations were quantified according to odds ratios (ORs) and 95% confidence intervals (CIs). The Hosmer-Lemeshow test was used to assess the validity of the model and the adequacy of the data; it validated the number of confounders included in our model and excluded multicollinearity. A composite endpoint was also defined, comprising the most severe outcomes including failure of the first course of ibuprofen, surgery, necrotising enterocolitis, intestinal perforation and use of dopamine or furosemide during the first course of ibuprofen. Dopamine was infused for persistent systemic hypotension and furosemide for persistent oliguria.

Statistical analysis was performed with SPSS V.15.0 (SPSS Inc.; Chicago, IL, USA).

## Results

### Characteristics of the study population

**Fig 1:**
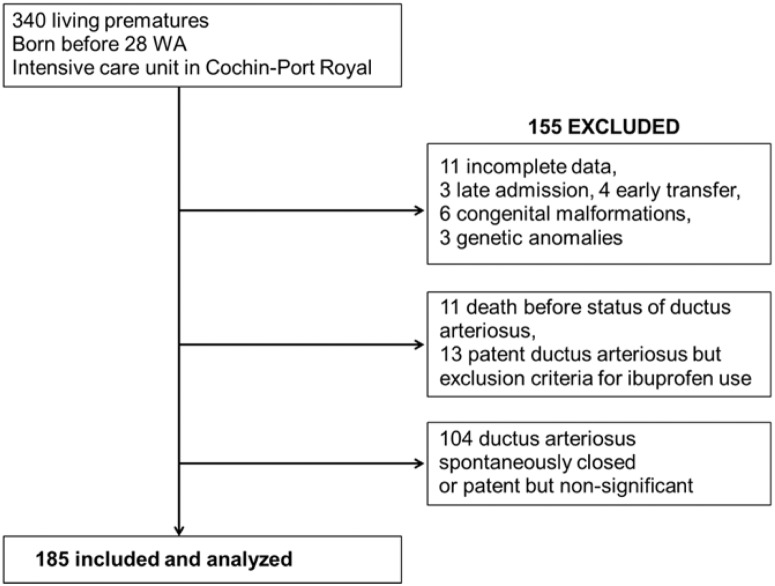
Patient flow.

This study included 185 patients (Fig 1). Fifty-four percent of eligible infants were born at 24+0–27+6 weeks of gestation, presented haemodynamically significant PDA, and were treated with at least one course of intravenous ibuprofen. Thirteen patients presented exclusion criteria for ibuprofen use; they presented grade 3 or 4 intraventricular haemorrhage, intestinal perforation, multiple organ dysfunction syndrome, or refractory hypoxia. When these 13 patients were included, at least 58% of the study population presented haemodynamically significant PDA. The GA-specific Z-scores for birth weight among the patients included were > -0.5 SD for 130 (70%) patients, -0.5 – -1.5 SD for 37 (20%) patients, and < -1.5 SD for 18 (10%) patients (Table 1).

**Table 1:** Baseline characteristics.

	MD		Total		> -0.5 SD		- 0.5 /- 1.5		< -1.5 SD		p
			185	(100)	130	(70)	37	(20)	18	(10)	
**Obstetric data**											
Multiple pregnancy	0	(0)	52	(28)	37	(29)	13	(35)	2	(11)	0.18
Outborn	0	(0)	13	(7)	12	(9)	1	(3)	0	(0)	0.18
PROM	0	(0)	55	(30)	46	(35)	9	(24)	0	(0)	0.01
AGC	1	(0.5)	140	(76)	92	(71)	31	(83)	17	(94)	0.05
Pre-eclampsia	0	(0)	35	(19)	5	(4)	13	(35)	17	(94)	<0.001
Chorioamnionitis	0	(0)	57	(31)	50	(39)	7	(19)	0	(0)	<0.001
Caesarean section	0	(0)	82	(44)	42	(32)	23	(62)	17	(94)	<0.001
**Neonatal data**											
GA (WA)	0	(0)	25.9	(1.0)	25.8	(1.0)	26.2	(0.9)	26.3	(0.8)	0.01
Male	0	(0)	101	(55)	70	(54)	19	(51)	12	(67)	0.50
BW (grams)	0	(0)	863	(176)	927	(161)	751	(94)	630	(68)	<0.001
BL (cm)	2	(1)	34.3	(2.2)	34.9	(2.0)	33.6	(1.6)	31.6	(1.5)	<0.001
BHC (cm)	9	(5)	23.8	(1.6)	24.2	(1.6)	23.3	(1.2)	22.6	(1.1)	<0.001
Lactate (mmol/L)	2	(1)	4.9	(3.2)	4.3	(3.0)	5.4	(3.1)	8.2	(3.2)	<0.001
Ventilation (days)	3	(2)	15.6	(15.1)	14.4	(16.3)	15.3	(11.6)	21.8	(10.4)	0.20
HFO (days)	0	(0)	1.7	(3.6)	1.4	(3.3)	0.8	(2.6)	5.2	(5.6)	<0.001
MFI	0	(0)	5	(3)	0	(0)	0	(0)	5	(4)	0.34*
Grade 3 or 4 NEC	0	(0)	13	(7)	11	(9)	2	(5)	0	(0)	0.38*
Intestinal perforation	0	(0)	11	(6)	9	(7)	0	(0)	2	(11)	0.18*
Grade 3 or 4 IVH	0	(0)	12	(7)	10	(8)	2	(5)	0	(0)	0.44*
BPD	28	(15)	105	(67)	63	(59)	26	(79)	16	(94)	0.01
PVL	4	(2)	6	(3)	6	(5)	0	(0)	0	(0)	0.28*
Death	0	(0)	18	(10)	14	(11)	1	(3)	3	(17)	0.20*

Data are expressed as number (%) or mean (SD: standard deviation).

*: p values were obtained with Fisher’s exact test (category 1 versus 2 and 3).

MD: missing data; PROM: premature rupture of membranes (>24 hours); AGC: antenatal glucocorticoids (at least two doses at an interval of 24 hours); GA (WA): gestational age (weeks of amenorrhoea); BW: birth weight; BL: birth length; BHC: birth head circumference; Lactate: baseline serum lactate; Ventilation: endotracheal ventilation; HFO: high frequency oscillatory ventilation; MFI: materno-foetal infection; NEC: necrotising enterocolitis (Bell classification); IVH: intraventricular haemorrhage (Papile classification); BPD: bronchopulmonary dysplasia; PVL: periventricular leukomalacia.

Review of obstetric data revealed no cases of chorioamnionitis for the most severely growth-restricted infants, but their mothers presented pre-eclampsia in about 95% of cases (Table 1). The study population was therefore relatively homogeneous, as intrauterine growth restriction was mostly secondary to placental insufficiency. Eighteen infants died (10%). No significant difference was observed between groups in terms of GA-specific Z-scores for birth weight (p = 0.20). Bronchopulmonary dysplasia was diagnosed in 105 patients (67%), and its frequency increased as the birth weight Z-score decreased (p = 0.01) (Table 1). The only significant difference among the patients excluded from the study was a higher GA (p = 0.01) (data not shown).

Higher baseline serum creatinine before initiating the first course of ibuprofen were observed in patients with lower GA. No statistically significant differences in terms of treatment characteristics were observed between the various birth weight groups (Table 2). Treatments were discontinued in the presence of contraindications to ibuprofen: severe haemodynamic failure, necrotising enterocolitis, intestinal perforation, grade 3 or 4 intraventricular haemorrhage, serum creatinine >120 μmol/L, platelet count <50,000/mm^3^, or clotting disorders. Serum creatinine and platelet count were determined before each dose of ibuprofen.

**Table 2:** Ibuprofen treatment characteristics.

	MD		Total		> -0.5 SD		- 0.5 /- 1.5		< -1.5 SD		p
			185	(100)	130	(70)	37	(20)	18	(10)	
Age at the beginning of treatment (days)	7	(4)	3.8	(2.9)	4.0	(3.1)	3.3	(2.5)	3.3	(1.7)	0.38
Serum creatinine before ibuprofen	3	(1.6)	81.1	(18)	79.7	(16.8)	80.5	(15.1)	91.8	(27.1)	0.03
2 courses initiated	0	(0)	73	(40)	49	(38)	16	(43)	8	(44)	0.50
3 courses initiated	0	(0)	7	(4)	7	(5)	0	(0)	0	(0)	0.50
Discontinuation of the first course	0	(0)	21	(11)	11	(9)	4	(11)	6	(33)	0.10*
Dopamine during ibuprofen therapy	0	(0)	8	(4)	4	(3)	1	(3)	3	(17)	0.37*
Furosemide during ibuprofen therapy	0	(0)	26	(14)	15	(12)	5	(14)	6	(33)	0.20*
PDA surgery	0	(0)	57	(31)	38	(29)	12	(32)	7	(39)	0.69

Data are expressed as number (%) or mean (SD: standard deviation). MD: missing data.

*: p values were obtained with Fisher’s exact test (category 1 versus 2 and 3).

### Primary outcome

In this study population, 136 patients experienced the most severe outcomes included in the composite criterion: 90 patients with Z-scores > -0.5 SD (69%), 29 patients with Z-scores -0.5 – -1.5 SD (78%), and 17 patients with Z-scores < -1.5 SD (94%). The risk of a severe outcome after a first course of ibuprofen therapy tended to increase as the patient’s birth weight decreased (with respect to their GA) (post-hoc comparison, p = 0.057).

The baseline characteristics of the infants in the three groups of GA-specific Z-scores for birth weight are summarised in Table 1. Potential confounders for the regression model were derived from univariate analyses of baseline characteristics. The most relevant data were analysed, i.e. data with a significant difference between the three groups (p <0.05): GA, premature rupture of membranes, antenatal glucocorticoid levels, pre-eclampsia, chorioamnionitis, and caesarean section. Baseline serum lactate levels were also included to take into account the patient’s initial adaptation to extrauterine life. Hosmer-Lemeshow tests confirmed the absence of multicollinearity, indicating an appropriate number of confounding factors for the sample size.

The first course of ibuprofen failed in 101 patients (55%) in this population. The relationship between failure of the first course of ibuprofen and SGA was studied by means of a logistic regression model (Table 3).

**Table 3:** Risk of failure of the first course of ibuprofen.

Gestational age-specific Z-score for birth weight and adjustment	OR [95% CI]	p value
**Crude OR**		
Z-score > −0.5 SD	1	
Z-score −0.5–−1.5 SD	1.4 [0.7–3]	0.33
Z-score < −1.5 SD	3.8 [1.2–12.3]	0.02
**Adjustment for GA, AGC, chorioamnionitis, PROM, pre-eclampsia, caesarean section, baseline serum lactate, and complete course of ibuprofen**		
Z-score > −0.5 SD	1	
Z-score −0.5–−1.5 SD	2.6 [1.03–6.8]	0.04
Z-score < −1.5 SD	12.8 [2.3–70.5]	0.003

GA: gestational age; AGC: antenatal glucocorticoids (defined by at least one complete course of two doses at an interval of 24 hours); PROM: premature rupture of membranes (>24 hours).

SD: standard deviation; OR: odds ratio; CI: confidence interval.

Regression analysis revealed an increased risk of failure of the first course of ibuprofen therapy for the smallest patients. Development of the regression model revealed a significant gradient: as birth weight for gestational age decreased, the risk of failure of a first course of ibuprofen increased. This association was expressed in terms of the OR in our logistic regression. OR values gradually increased as variables were progressively added to the regression model, demonstrating that the more the logistic regression was adjusted, the more a low Z-score influenced the risk of failure of the first course of ibuprofen.

## Discussion

To the best of our knowledge, this is the first study to describe the impact of SGA on the treatment of haemodynamically significant PDA in extremely preterm infants. This cohort included 185 infants, 55 of whom had a GA-specific Z-score for birth weight < -0.5 SD. The risk of failure of the first course of ibuprofen increased with the degree of growth restriction, reaching a maximum 12.8-fold higher risk of failure, according to a gradient that intensified with regression adjustments.

Birth weight SDs were classified into three categories to reflect the continuous pathological effect of being small-for-gestational age on mortality. This classification avoided a cut-off effect and revealed a gradient for the primary outcome, supporting the validity of the main results and regression models. Even if, at first, epidemiologic studies described preterm infants growth restriction using a −2 SD (or 10th centile) cut-off for SGA, more recent studies proved that this definition is no more appropriate because it inadequately describes the risks associated with foetal growth restriction [3,5].

One of the strengths of this study was the definition used for significant PDA, based on precise echocardiographic criteria. All data were collected prospectively as part of a routine neonatal care and were regularly validated by the clinical team, resulting in fewer missing data and the collection of more reliable data. Treatment protocols in the department did not change during the study period. The dose regimen complied with the *Haute Autorité de Santé* (French health authority) guidelines, based on pharmacological data [12,13], and validated in a double-blind dose-finding study using the continual reassessment method [14].

Previous studies only used birth weight, unrelated to GA, to analyse the effect of ibuprofen therapy [15]. Studies on PDA management were not designed to include SGA subgroup analysis, but have often analysed SGA as a risk factor for PDA, rather than a predictive factor for treatment failure.

The overall incidence of haemodynamically significant PDA treated with at least one course of ibuprofen in our population was 54%. Our descriptive analyses are consistent with those reported in the literature; this rate was comparable to that observed in infants weighing <1 kg, 55% of whom received medical treatment for PDA [9]. The overall failure rate for the first course of ibuprofen in our study was 55%. This rate is higher than those reported in the literature, ranging from 20 to 40% [16]. Sixty-two percent of infants in our study were born before 27 weeks of gestation; in previously published studies, infants were born at gestational ages of up to 34 weeks [8]. This discrepancy could account for these differences in patent ductus arteriosus closure rates. Our closure rates are comparable to those reported in the study by Desfrère et al., who used the same dose regimen in a similar population [14] and who reported a 30% closure rate 24 hours after the first course of ibuprofen in infants born before 27 weeks of gestation.

Multiple mechanisms contribute to patent ductus arteriosus closure [17]. These mechanisms can offset each other, which could explain the incomplete efficacy of prostaglandin inhibitors. However, less is known about how prematurity and SGA deregulate these pathways and the effect of ibuprofen therapy.

Our descriptive analyses highlighted the confounding variables used to build our regression models, and the Hosmer-Lemeshow test demonstrated the validity of our models and partly excluded multicollinearity. The regression analysis showed that the association between growth restriction and failure of the first course of ibuprofen was independent of the confounding variables (GA, obstetric data, baseline serum lactate levels, and discontinuation of the course of treatment).

The first course of ibuprofen tended to be discontinued more frequently in infants with GA-specific birth weights < -1.5 SD, probably because of the more cautious attitude adopted by the medical team in these patients. This criterion was entered into our regression model to control for the potential confounding effect of variations in the approach adopted by the clinicians.

Despite the low statistical power of this study, due to the limited sample sizes, especially for the smallest infants, statistical analysis supported the results in this homogeneous population, as this study is one of the few examining birth weight Z scores in extremely preterm infants and PDA treatment as the main outcome.

This lack of power resulted in large confidence intervals; however, the main results demonstrated at least a 2-fold risk of treatment failure.

The results of this study support the hypothesis that PDA treatment strategies in very preterm infants may be adapted to their birth weight SD. Previous clinical trials have speculated that increasing the dose of ibuprofen may increase PDA closure rates [14,18,19]; however, these results are controversial in view of the increased toxicity of ibuprofen in these patients. Future studies should investigate whether earlier or longer treatment may increase ibuprofen efficacy for the most premature and growth-restricted infants.

In conclusion, we analysed the effects of ibuprofen with respect to birth weight for gestational age. This constitutes an original approach, as previous studies have assessed birth weight or gestational age without considering small-for-gestational age patients. Our results indicate that PDA treatment strategies in very preterm infants may be adapted to their birth weight standard deviation, as this study showed decreasing birth weight standard deviations were associated with higher failure rates of the first course of ibuprofen. These results need to be confirmed in larger, multicentre cohorts. However, we suggest that prospective studies on the PDA closure therapeutic strategies in extremely preterm infants should be analysed according to GA-specific birth weight subgroups.
